# Effectiveness of Gel Repellents on Feral Pigeons

**DOI:** 10.3390/ani4010001

**Published:** 2013-12-19

**Authors:** Birte Stock, Daniel Haag-Wackernagel

**Affiliations:** Department of Biomedicine, University of Basel, Pestalozzistrasse 20, 4056 Basel, Switzerland; E-Mail: daniel.haag@unibas.ch

**Keywords:** capsaicin, *Columba livia*, contact gel, feral pigeon, optical gel, repellent gel

## Abstract

**Simple Summary:**

Feral pigeons live in close association in urban areas. They constitute serious health risks to humans and also lead to high economic loss due to costly damage to buildings, historic monuments, statues and even vegetation. While numerous avian repellent systems are regularly introduced onto the market, scientific proof of efficacy and their use from the point of view of animal welfare is lacking. Therefore, two avian gel repellents were studied on free-living feral pigeons in this study. The focus was set on repellent efficacy and animal welfare concerns. This study’s aim is to contribute to a better understanding of feral pigeon management in our cities.

**Abstract:**

Millions of feral pigeons (*Columba livia*) live in close association with the human population in our cities. They pose serious health risks to humans and lead to high economic loss due to damage caused to buildings. Consequently, house owners and city authorities are not willing to allow pigeons on their buildings. While various avian repellents are regularly introduced onto the market, scientific proof of efficacy is lacking. This study aimed at testing the effectiveness of two avian gel repellents and additionally examined their application from animal welfare standpoint. The gels used an alleged tactile or visual aversion of the birds, reinforced by additional sensory cues. We mounted experimental shelves with the installed repellents in a pigeon loft and observed the behavior of free-living feral pigeons towards the systems. Both gels showed a restricted, transient repellent effect, but failed to prove the claimed complete effectiveness. Additionally, the gels’ adhesive effect remains doubtful in view of animal welfare because gluing of plumage presents a risk to feral pigeons and also to other non-target birds. This study infers that both gels lack the promised complete efficacy, conflict with animal welfare concerns and are therefore not suitable for feral pigeon management in urban areas.

## 1. Introduction

The feral pigeon, the descendant of the domesticated form of the wild living Rock Dove (*Columba livia*), is a highly successful urbanophilic species, which occurs worldwide. With a domestication history of several thousand years [1], feral pigeons are well adapted to human environments. Due to the abundant feeding options in our cities, feral pigeons have expanded their originally granivorous diet to an omnivorous one [2]. In addition to the positive nutritional effects, cities with house facades, churches and statues offer an ideal environment for the birds. Pigeons that originally lived along coasts with cliffs now use numerous structures associated with urban buildings as roosting, resting, nesting and outlook spots. The close association of large feral pigeon populations and humans creates a human-wildlife conflict with serious health risks. With more than 100 human pathogenic microorganisms and 18 ectoparasites associated with feral pigeons [3,4], the epidemiological significance of these birds to humans is evident. Although the risk of zoonotic diseases caused by feral pigeons is rare, fatal cases have been reported [5]. Besides the medical risk, feral pigeons living in urban habitats also lead to high economic loss due to significant damage to buildings, historic monuments, statues and even vegetation [2]. The removal of pigeon droppings from buildings causes high costs [6]. With an individual pigeon producing around 4–11 kg of excrement each year [7], enormous quantities of pigeon droppings end up in every larger city of the world. This excrement offers a substrate for the growth of microorganisms that are able to destroy building materials [8]. 

In addition to these negative esthetic and hygienic aspects, the costs of feral pigeons living in urban environments are high. The estimated damages per feral pigeon per year including pollution of buildings, streets and places, as well as hygienic costs, agricultural costs and bird strikes range from 23.7€ to 33.5€ [9], which equals approximately $US 31 to 44. In the USA, the damage caused by feral pigeons has been estimated to $US 1.1 billion per year, not including environmental damage associated with the pigeons serving as reservoirs and vectors for diseases [10]. The relevance of pigeons is further pointed out by the number of about 22’500’000 hits when entering the words “pigeon problems” into the internet search engine Google (accessed 28 October 2013).

Frequently recommended solutions to solve the pigeon problems in residential areas and city centers include a large number of nonlethal systems that repel and exclude the birds from buildings and monuments. Repellents can be used to manipulate animal behavior in a way that an animal is motivated to avoid the consequences of the aversive signal [11]. In general, animal repellent systems can be of visual, acoustic, tactile, olfactory, or gustatory nature, or even combine several of these characteristics [11,12,13,14,15,16,17]. The business of production and installation of avian repellent systems involves the sales of millions of dollars worth of products in Europe and the USA [9,18,19]. While netting and other exclusion systems are successfully used against pigeons, these methods do not always seem to be an economic or practical option [20], and such eye-catching systems often distract from the architectural impression [21]. In particular, historic buildings are sensitive to pigeon droppings and difficult to protect from these birds. With the sheltered niches, crevices and ledges common to ornamental facades, such buildings offer ideal nesting and roosting habitats [22]. Several other proofing products promise an optimal integration in the esthetic impression of building facades since they are inconspicuously and discretely mounted onto the affected structure or area. Whereas for example netting and spikes repel the pigeons on the basis of exclusion via mechanical barriers, other innovative systems are often supposed to work with aversive cues that motivate the bird to avoid the treated spaces. These new systems, which are regularly introduced onto the market, promise to be the ideal solution to the problems caused by pigeons on buildings. They are supposed to be not only effective, but also inconspicuous, easy to mount and available at a competitive price. However, data to support the expected results of these new, inventive and allegedly persistently effective bird repellents is rare or inexistent. Furthermore, these new products have rarely been put to test under the point of view of animal welfare. Given the fact that highly motivated pigeons are able to overcome almost every system [19], the effectiveness of new bird repellent products should be investigated critically.

A reasonable feral pigeon management in urban areas requires very good knowledge of proofing and scaring systems and the reactions of the birds towards them. We therefore tested two nonlethal, food-grade, avian repellent gels that are supposed to combine an easy and discrete installation with 100% success in removing the birds from treated areas within less than a week. While one gel is based on the alleged tactile aversion of the birds to capsaicin, the other claims to function through a visual repellent effect that is reinforced by ingredients that are repulsive to the olfactory, gustatory and tactile senses of the birds. 

The objective of our study was to assess the effectiveness of these two avian gel repellents by analyzing the behavior of feral pigeons when confronted with them. In addition to the efficacy of the products, we also focused on the gels from the point of view of animal welfare.

## 2. Materials and Methods

### 2.1. Study Area

We conducted our study in the pigeon loft of the St. Matthew Church, which is situated in a residential district of Basel, Switzerland (47.5671°N, 7.5930°E). The city of Basel is located in northwestern Switzerland, at the intersection of Switzerland, Germany and France. In August 2012 it counted around 170’000 inhabitants. The climate is continental and during the study period, average temperatures ranged from 20.7 °C in August to 10.7 °C in October.

The pigeon loft was situated above the nave of the church at a height of about 18 m above ground. Besides a floor space of 28 m^2^, the loft had 39 nesting boxes and several roosting bars. We set a timer for constant diurnal rhythm of 9 hours and 30 minutes of light and 14 hours and 30 minutes of dark in the loft. The experiments were performed under natural conditions without offering any food or water. The pigeons used the loft exclusively for roosting and breeding. Their food was generally foraged in the surrounding area and the city [23].

### 2.2. Tested Bird Repellent Gels

Two avian repellent gels were tested on free-ranging feral pigeons: a contact gel and an optical gel. Both products are used in pest bird management programs to protect structures from birds. Since repellent products are continuously changing their names or reentering the market only slightly modified, we refrain from providing the names of the products and the manufacturers. Instead, the tested products stand for a specific but conventionally used kind of repellent system. 

#### 2.2.1. Contact Gel

As specified by the manufacturer, the contact gel included non-toxic, 100% natural ingredients and can be used to protect all kinds of indoor and outdoor surfaces of buildings, monuments and also statues against nuisance birds, especially pigeons. The gel contained 0.0357% capsaicin, which is the pungent element of red pepper [24]. According to the distributor, capsaicin causes a mild harmless irritation when being transferred onto the feet of the birds by landing on the treated areas. This sensory reaction to the gel is supposed to condition the pigeons to avoid the location. The clear, odorless and semi-solid gel was supplied in 300 mL cartridges and applied on the experimental shelves in a wave pattern at a stretch according to the application instructions. The distributor claimed that 100% of the bird population would be successfully removed within seven days of gel application, which was allegedly proven during rigorous testing carried out by the developers.

#### 2.2.2. Optical Gel

The second bird repellent, which was examined, was an optical gel, sold by another distributor. According to the general product information, the gel is patented and contains food-grade natural oils. It is supposed to repel all birds from all indoor and outdoor structures without causing any harm to target animals. Ingredients in the product include polyisobutylene, grease lubrication, peppermint oil and cinnamon oil. According to the distributor, the gel is able to repel the pigeons visually because it is perceived as fire within the ultraviolet visual range of the birds. Furthermore, the distributor claimed that natural oils, which should be abhorrent to a bird’s senses of smell, taste and touch, reinforce the visual repellent effect. The gel was delivered in 250 mL cartridges with supplementary application dishes of 7 cm in diameter. We applied 15 g of the repellent gel in each dish as recommended in the manufacturer’s guidelines. 

After consultation with the distributor who determined the number and location of dishes on the experimental shelves, we arranged eight dishes per shelf in two parallel rows of four dishes. The dishes covered a total of 17% of the shelves. The greatest distance between two dishes was 13 cm. According to the application guide, this distance referred to an area with high bird density. The manufacturer claimed that after two or three days even the most dominant birds would avoid the treated areas.

### 2.3. Study Animals

The feral pigeon colony used for this study contained about 85 birds with an average body weight of 322 g. Due to the fact that the pigeon loft was freely accessible to every feral pigeon in the surrounding area and the birds of our study were able to enter and leave the loft at will, fluctuation of the population was possible. We routinely caught, ringed and weighed the resident pigeons every six months. During the study period, one pigeon that hatched in the loft became integrated into the population, another adult pigeon immigrated and six pigeons, both adults and young, left the population. Due to the periodical flock controls and the cleaning of the pigeon loft twice a month, the pigeons were habituated to human presence. Even though all pigeons of the loft were ringed, either directly as nestlings or as immigrated adults, the small ring numbers were not recognizable on the video material. An unambiguous assignment of the observed reactions of the pigeons to a particular bird was thus not performed.

### 2.4. Experimental Design and Data Collection

We installed four experimental shelves of 0.6 m length and 0.3 m width as resting, roosting and outlook spots for the pigeons in the loft. Each shelf was attached onto the wall at right angles, offering the birds a convenient area to perch. The shelves were placed in a zigzag pattern at heights of 0.8 m to 1.6 m, about 1.3 m away from the nesting boxes on the adjacent wall. After the installation, the pigeons were given ten days to get used to the new structures in the loft. We performed our experiment in August–October 2012. It consisted of two main phases: a pretrial of 16 days and a trial phase of 26 days. We monitored the experiment with a video camera (JVC model GY-HM150E, Yokohama, Japan) at random dates each for 24 hours. On 27 August 2012, we started the pretrial phase during which we video recorded three out of 16 days in a weekly rhythm to get a base value for the daily use of the shelves without the installed repellents. The dishes in which the optical gel was applied were not mounted during the pretrial phase. The idea was to first create a natural scene with an ordinary structure frequently used by pigeons and not treated with any kind of repellent or uncommon system. Each of the gels was applied on two of the experimental surfaces, according to the distributor’s guidelines, on 12 September 2012. However, the shelves and the wall on to which they were installed were thoroughly cleaned before application, as the products are said to only have full effectiveness when used on unsoiled structures, free from any bird excreta. We recorded 16 days of our 26 days trial phase, with the last recorded day being trial day 26. Due to methodological considerations, we eliminated the first trial day of the visual gel testing and restarted the experiment on the second day of recording. As a result, we excluded the first trial day from statistical analyses and assigned the actual second trial day as the first. Thus, the last recorded day of the visual gel testing was trial day 25.

In addition, the emissions and the lifetime of the excited states of the optical gel was measured as it is supposed to be perceived as fire within the ultraviolet visual range of the feral pigeons. The measurements were taken with the compact fluorescence lifetime spectrometer Quantaurus-Tau C11367-11 by Hamamatsu excited at a wavelength of 280 nm.

### 2.5. Animal Welfare Point of View

We conducted the experiments with the animal experimental permission of the Cantonal Veterinary Office of Basel-Town, Switzerland (authorization No. 2296). The study conformed to Swiss law on animal welfare. The permission allows experiments on animals causing mild stress, which corresponds to the severity Grade 1. According to Swiss animal welfare, severity Grade 1 studies include interventions and manipulations on animals for experimental purposes, which subject the animals to a brief episode of mild stress (pain or injury). Furthermore, it is claimed in Article 4(2) of the Swiss Animal Welfare Act that no person may, without justification, inflict pain, suffering, or injury upon an animal or cause it fear, or disregard the dignity of the animal in any other way. With this in mind, we first tested the pigeons’ behavior towards the gels applied in nesting boxes during a test run. During this test run, the pigeons entered their nesting boxes in all cases. Apparently, the birds were not repelled by the gels due to their high motivation to repossess their breeding places. Furthermore, because the chances of nestlings and inexperienced juvenile birds getting into contact with the sticky gels were too high, the nesting boxes test run was canceled prematurely. For that reason we chose to test the repellent gels on new, rather unpopular, experimental shelves in heights starting at 0.8 m so that nestlings and badly flying juveniles were not able to smear the sticky products into their not yet fully grown plumage. With these low motivation structures, not being as fiercely contested as other areas in the loft, the risk of gluing of plumage of adult pigeons was further minimized. 

### 2.6. Data Analysis

We evaluated the recorded behavior and analyzed the number of approaches and landings, as well as the time spent on the experimental shelves prepared with the two repellents for each recorded day. A successful repellent system reduces the number of birds using the protected structure by 100%. Although a general reduction might seem effective to non-experts, only a complete protection marks a successful repellent system. Even low numbers of pigeons still using and soiling the treated areas point out the failure of the repellent system. For the simple reason that even a single pigeon is able to transmit human pathogenic diseases, a repellent system should not only reduce the number of pigeons using a treated structure, but completely remove the birds from it. Due to this reason, the success of the repellents was determined as a reduction of feral pigeons’ use of the experimental shelves by not less than 100%.

Based on the claim of the contact gel distributor, complete avoidance of the prepared shelves was to be expected within seven days of gel application. We therefore categorized three trial phases: pretrial (three recorded days), trial Days 1–7 (five recorded days) when full effectiveness was not yet expected and trial Days 8–26 (11 recorded days) when complete effectiveness was anticipated. 

For the visual gel we similarly analyzed the number of approaches and landings, together with the time spent on the shelves. The distributor of the visual gel claimed that the product would be absolutely effective within three days of product application. We characterized three trial phases: pretrial (three recorded days), trial Days 1–3 (two recorded days) and trial Days 4–25 (13 recorded days). Additionally, we distinguished between different behaviors of the pigeon towards the visual repellent: (a) approach without landing and therefore no possible contact, (b) landing with immediate gel contact, (c) subsequent gel contact, and (d) no contact with the gel. We combined the data from the two shelves with the same repellent due to the vicinity of the shelves.

The statistical tests were carried out with the open source statistical package R (R Version 2.15.1 and for the residual analyses R Version 3.0.1 for Mac).

The number of approaches per day for both gels was analyzed using a Quasi-Poisson model (function glm) with phase (three levels as described above) as the sole explanatory factor. Quasi-Poisson was used to account for overdispersion of the data. To model the time spent on the shelves per landing for each gel, we used a linear mixed model (function lmer) with the log-transformed time spent on the shelves as the outcome variable, phase as fixed factor and day as random factor. As uncertainty intervals we calculated Bayesian 95% credible intervals based on 5,000 simulations from the posterior distribution for both number of approaches and time spent on the shelves. Residual analyses included visual inspection of residual versus fitted values plots, quantile-quantile plots for both random effects and fixed effects residuals, as well as temporal autocorrelation plots. These plots indicated no serious violation of model assumptions and no substantial autocorrelation. We use the term “significant” for a fixed effect when the fitted value of one level is not included in the 95% credible interval of the other level.

Moreover, except for the approach without landing, we subdivided the possible behaviors relating to the contact of the landing pigeon with the visual gel (immediate contact, subsequent contact or no contact) into two time based categories: time spent on the experimental shelf ≤3 seconds, or >3 seconds. As pigeons have short reaction times of less than half a second, even in multi-option experiments [25], the 3 seconds that were set as the time to react to the repellents were generously determined and in favor of the effectiveness of the gels. Due to the fact that the complete repellent effect of the visual gel is supposed to have developed two or three days after gel application, we only included trial Days 4–25 in the evaluation of the affected senses. The distributor stated that the optical gel would influence the behavior of the pigeons by affecting not only the visual sense of the birds, but also the senses of smell, touch and taste. We therefore categorized the behaviors of the pigeons into seven classes to determine the affected sense in case of a positive repellent effect. We set the distant visual sense as being influenced when a pigeon approached the shelves but did not land on them. Stimulus of the near visual sense was given if the pigeon left within ≤3 seconds after it had landed on the experimental shelf and showed immediate or no contact with the gel. We defined no visual repellent effect if the pigeon landed first and had subsequent contact with the gel. For the olfactory sense we also set 3 seconds as the time between contact and flying away as the limit for a successful repellent effect, except for the subsequent contact category. Here we defined the inefficacy of the olfactory repellent effect if a pigeon landed on the shelf first and stepped into the gel afterwards. We defined a failure of the system in a tactile sense if the pigeon stood for >3 seconds in the gel. Due to the rare occurrence of events in these categories, a statistical analysis of these data was not appropriate but results were compiled in Table 1.

In terms of the animal welfare point of view we observed the consequences of the pigeons having direct contact with the gels. In addition, the effect of the gel remains transferred to other structures in the loft, and possibly also outside the loft, was described with the potential consequences for other birds.

## 3. Results

### 3.1. Contact Gel

**Figure 1:**
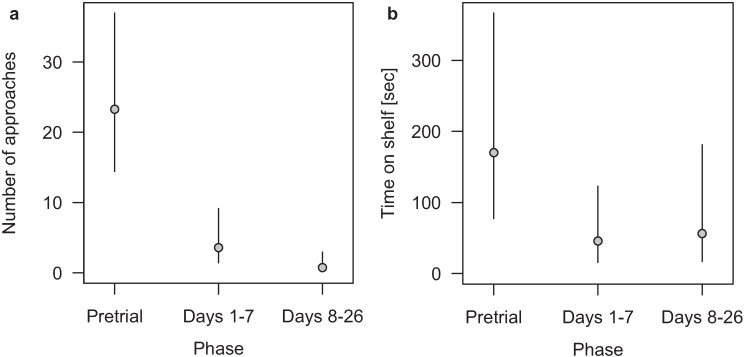
Feral pigeons’ (**a**) mean number of approaches per day and (**b**) mean time spent on the shelf in seconds per approach for the three phases pretrial, Days 1–7 and Days 8–26 of the contact gel experiment in Basel, Switzerland, during August–October 2012. Values are means and the segments indicate Bayesian 95% credible intervals. For the mean number of approaches, with n per phase being 3, 5 and 11 recorded days, respectively, a Quasi-Poissonmodel was used. For the mean time spent on the shelf a mixed model with the log-transformed time on the shelf as the outcome variable (results back transformed for the graph) phase as fixed factor, and day as random factor was used with n per phase being 70, 18 and 8, respectively.

Figure 1(a,b) shows the results of the contact gel experiment. The numbers of pigeon approaches to the shelves differed by phases. The highest number occurred to the shelves without repellent gel during the pretrial phase (70 approaches). We noted less approaches throughout trial Days 1–7 (18 approaches) and the least during trial Days 8–26 (eight approaches). During the pretrial phase, a mean of 23.3 approaches per day (14.4–37.0 Bayesian 95% credible interval), during trial Days 1–7 a mean of 3.6 (1.4–9.2) and during trial Days 8–26 a mean of 0.75 (0.18–2.95) approaches per day were recorded. The time spent on the experimental shelves during pretrial phase was significantly (or near significantly) longer than during both of the trial phases, but no significant difference occurred between trial Days 1–7 and 8–26 (Figure 1b). During the pretrial phase, the pigeons spent a mean time of 170 (77–367) seconds per landing on the shelf. Trial Days 1–7 showed a mean of 46 (16–123) seconds and trial Days 8–26 a mean of 56 (17–181) seconds per landing. Moreover, we observed only one approach during the pretrial phase that did not lead to a final landing. At this occasion the pigeon flew in the direction of an experimental shelf but turned away shortly before reaching it. In contrast, during trial phase all approaches led to a landing. 

### 3.2. Optical Gel

**Figure 2:**
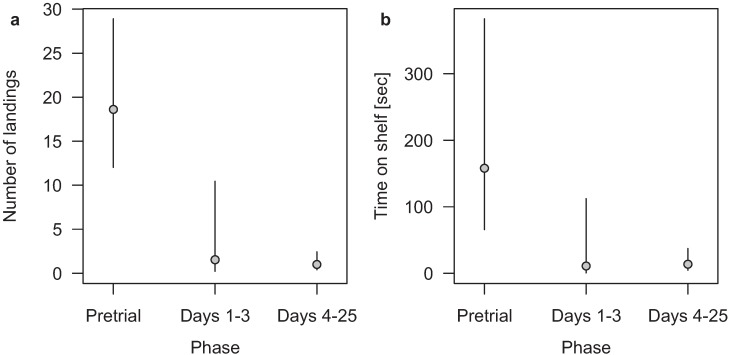
Feral pigeons’ (**a**) mean number of landings per day and (**b**) mean time spent on the shelf in seconds per landing for the three phases pretrial, Days 1–3 and Days 4–25 of the optical gel experiment in Basel, Switzerland, during August–October 2012. Values are means and the segments indicate Bayesian 95% credible intervals. For the mean number of landings, with n per phase being 3, 2 and 13 recorded days, respectively, a Quasi-Poisson model was used. For the mean time spent on the shelf, a mixed model with the log-transformed time on the shelf as the outcome variable (results back transformed for the graph), phase as fixed factor, and day as random factor was used with n per phase being 56, 3 and 13, respectively.

During the optical gel repellent test we observed that all approaches to the experimental setup were finished with a landing. We observed a total of 56 landings during the pretrial phase. For trial Days 1–3 we monitored a total of three landings and for trial Days 4–25 a total of 13 landings. The trial phase showed a significant decrease in landings per day compared to the pretrial phase (Figure 2a). During the pretrial phase we detected a mean of 18.6 (12.0–28.9) landings per day, during trial Days 1–3 a mean of 1.53 (0.23–10.45), and during trial Days 4–25 a mean of 1.01 (0.40–2.44). We recorded no difference between trial Days 1–3 and 4–25.

Figure 2b shows that during the pretrial phase, when the shelves were not prepared with the optical gel, the pigeons spent significantly more time on the shelves per pigeon landing than during the trial phases. We observed a mean time spent on the shelves per landing of 158 (66–383) seconds during the pretrial phase, a mean of 11 (0.4–112) seconds for trial Days 1–3 and a mean of 14 (4.5–37) seconds for trial Days 4–25. There was no significant difference between the two trial phases.

We summarized the behaviors of the pigeons to the optical gel during trial days 4–25 into seven categories to analyze which sense could have been influenced by the aversive signal (Table 1). All observed 13 approaches led to a landing and all of the stays on the protected shelves lasted >3 seconds.

**Table 1:** Number of behavioral responses of feral pigeons to the tested optical gel on trial Days 4–25 with determination of the senses appealed to in Basel, Switzerland, during August–October 2012. *f*, far; *p*, possible.

Behavioral response	*n*	Appealed senses
Approach without landing	0	Visual (*f*)
Landing, immediate contact, ≤3 sec	0	Visual, tactile, olfactory
Landing, immediate contact, >3 sec	7	No visual, no tactile, no olfactory
Landing, subsequent contact, ≤3sec	0	No visual, tactile (*p*), no olfactory
Landing, subsequent contact, >3 sec	4	No visual, no tactile, no olfactory,
Landing, no contact, ≤3 sec	0	Visual, olfactory
Landing, no contact, >3 sec	2	No visual, no olfactory

When testing the emission of the optical gel, a maximum at 357 nm was found. This demonstrates that the product did emit in the ultraviolet light range, which covers wavelengths of 100 nm until 380 nm.

**Figure 3:**
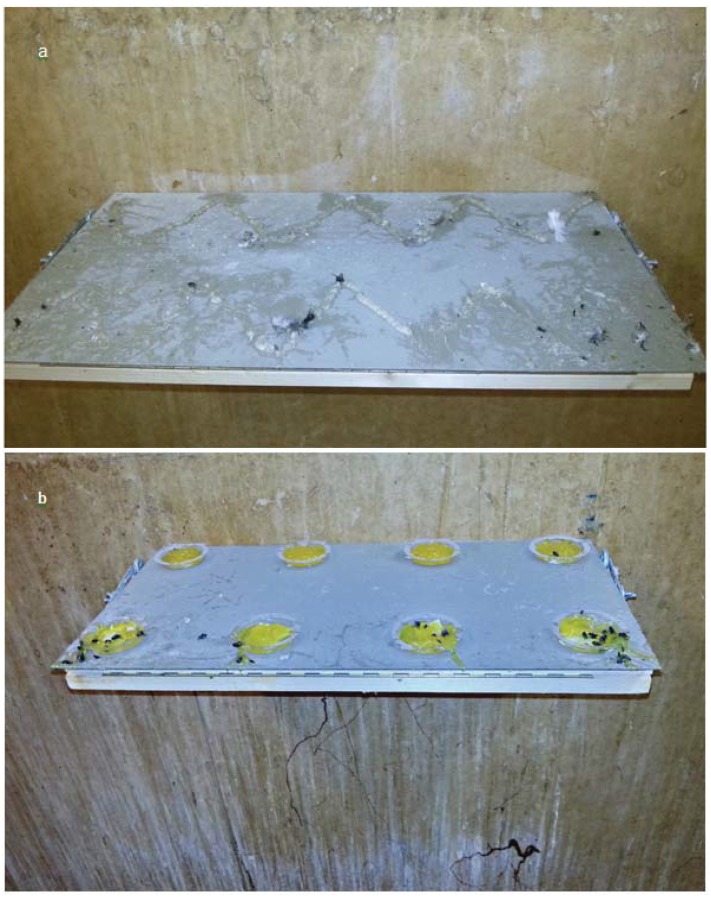
Appearance of the tactile gel (**a**) and the optical gel (**b**) after 23 days of application. Due to the adhesive effect numerous insects, feathers, dust and feces became stuck in the gels. The gluey optical gel got stuck on the wall underneath the experimental shelf when the pigeons stepped into the repellent and flew off pulling long adhesive strings. These remains were extremely difficult to remove.

As to the animal welfare point of view we could observe several pigeons stepping into the gels, either directly when landing onto the experimental shelves or subsequently after landing next to the shelves. Already after a short period of time, both gels looked rather unesthetic and messy due to a variety of insects, feathers and dirt that become stuck in the repellents either directly or in the remains on the shelves (Figure 3). 

While the tactile gel is rather harmless to pigeons regarding its stickiness, the optical gel is of extremely adhesive texture. Here, the possibility of gluing of plumage is definitely given. In addition, it was observed that birds transferred the gels, especially the optical one, to numerous other structures into the loft. Due to the extremely gluey structure of the optical gel, the birds pulled long strings when they stepped into the gel and flew off (Figure 3b). These strings got stuck not only to the experimental shelves, but also to the walls, the ground and were transferred to divers other areas in the loft, as for example the nesting boxes. We can not ensure that the gel was being transferred to other areas outside the loft, but this option seems likely when looking at the numerous traces of gel being spread all over the loft. When cleaning the loft, it was extremely difficult to entirely remove the gel remains. Even strong cleaning agents were used, but some adhesive residues could not be completely removed. 

## 4. Discussion

Both gels showed a restricted repellent effect by reducing the number of approaches of feral pigeons and their time spent on the experimental shelves per landing, but the claimed complete effectiveness, meaning a reduction of the number of birds using the protected structures by 100%, was not observed.

### 4.1. Contact Gel

The number of approaches during the contact gel experiment decreased constantly over the trial phases. The time spent on the shelves decreased initially, but increased again slightly during trial Days 8–26. We suspect this could be due to initiating habituation. The chance of new birds entering the loft was very low. Techniques such as tactile repellents are recognized to be of limited use because the learned avoidance of the unpleasant sensation extinguishes rapidly [11]. The repellent mechanism of the product tested is supposedly based on a slight irritation of the birds by means of capsaicin, the pungent element of red pepper. While capsaicin is an extremely effective irritant for mammals, birds are almost totally insensitive to it [13,15,16,26,27]. For this reason, a claimed sensory reaction to the gel, as stated by the distributor, is not expected. Instead, we attribute the observed repellent effect as a result of neophobia and discomfort. No complete avoidance of the experimental shelves was observed after a week of gel application. The pigeons rather appeared to get used to the new substance. They often flew onto the treated surface and stood in the repellent, which led to a constant removal of the gel (Figure 3a). Due to this contact with the gel, feces, dirt and feathers were regularly transferred onto the experimental shelves, masking any tactile effect. In addition, numerous insects also became stuck in the gel. Even though the sticky effect of the tactile repellent did not appear to be dangerous for the pigeons, any adhesive effect would make the gluing of plumage possible [12] and therefore contradicts animal welfare. When the birds preen themselves, they possibly disperse the gel even further over and into their plumage. The gel can also be transferred onto other structures and potentially affect non-target, perchance even protected, species. 

A repellent effect was detected, but a complete effectiveness of the gel, which is necessary in feral pigeon proofing, is missing. Additionally, the gel has an unpleasant esthetic aspect and a limited life span due to fouling with dust, insects, feathers and feces. Furthermore, the possibility of gluing of plumage and of affecting other structures and non-target birds is given. Due to these reasons, we cannot recommend the tested tactile gel repellent.

### 4.2. Optical Gel

The optical gel repellent led to a decrease in landings over the trial phases. The time spent on the experimental shelves per landing was initially reduced but then increased again slightly during trial Days 4–25. The gel failed to achieve complete effectiveness since the pigeons still flew onto the treated surfaces after more than 3 days of gel application. According to the distributor, the product tested is able to repel birds visually because it is perceived as fire in their ultraviolet visual spectrum. In addition, the distributor claimed a reinforced repellent effect caused by natural oils that should be abhorrent on an olfactory, gustatory and tactile basis. Even though the effectiveness of certain repellents can be improved by additional sensory cues [28], this gel did not achieve complete avoidance of the perch area after three days of application and thus failed to prove the essential full effectiveness. According to the distributer’s statement, the gel is seen as fire by the birds. Despite the fact that pigeons are certainly sensitive to ultraviolet light [29] and therefore could possibly perceive the gel as fire, one wonders how a pigeon should be familiar with fire and associate it with danger given the lack of experience. An inborn avoidance of ultraviolet light and fire lacks any evidence. The emission measurement of the optical gel showed that the gel did emit in the ultraviolet light range. However, only flames at temperatures hotter than 2,500 °C contain ultraviolet parts of the light spectrum. A normal fire by contrast does not contain ultraviolet light [30]. The reasoning of the birds seeing the optical gel as fire could therefore not be reconstructed. In addition, the effect of an outdoor use of the gel in the dark, as well as an indoor use without a supplementary artificial light source, remains questionable. According to our tests, it is not possible that the optical gel owns a repellent effect due to ultraviolet light emission. We suggest instead that the observed change in landings and time spent on the experimental shelves is due to other factors.

Furthermore, we observed a unique event during which a pigeon landed directly into one of the dishes and pecked into the gel repellent after about two seconds. This was repeated twice 13 seconds later. This observation suggests that the gel has no negative effect on the gustatory sense of a pigeon. In addition, all of the 13 approaches led to a landing and the pigeons stood longer than three seconds on the protected shelves or even directly in the gel. This further suggests that the gel does not work on the above-mentioned senses of pigeons.

## 5. Conclusions

Overall, we conclude that both gels showed a repellent effect, but failed to display the complete effectiveness that is unquestionably essential for a successful feral pigeon management. Our results indicate that capsaicin is ineffective in feral pigeon repellent systems. This is consistent with several other studies and the fact that pigeons are not irritated by capsaicin due to their lack of capsaicin-receptors [13,15,16,26,27]. The primarily observed repulsive effect of both gels is presumably due to neophobia, discomfort and the reduction of space on the shelves. For our second trial phase, we observed a slight, yet statistically not significant, increase in the time spent on the shelves per landing for both gels. Such a fading effect of the repellent is most likely to occur if this effect is based on startle responses due to neophobia. If the relevant stimuli are presented more than a few times, the animals desired to be repelled get accustomed to them [13,31]. 

As previously shown [19], young and inexperienced birds in particular landed repeatedly on the protected structures. Thus a test run was cancelled prematurely because the chance of nestlings getting directly into contact with the gels was too high. Especially the optical gel had an extreme adhesive effect, which could possibly lead to severe gluing of plumage of any bird as it already occurred with other so-called safe bird repellents [32]. Even weeks after the end of the study, we detected sticky remains of the repellents in the loft. This would definitely leave negative esthetic residues on surfaces if applied onto building facades, possibly causing even more damage than the pigeon droppings themselves. Given the possibility of young birds and also non-target birds coming into contact with the adhesive gels and the fact that any stickiness, even if relatively harmless, contradicts animal welfare, we can not approve the gels. 

In our experimental situation, the treated shelves were not particularly attractive to the birds because the pigeon loft offered enough room where the repellents could be avoided. The fact that the pigeons still landed on the treated surfaces shows that even pigeons with low motivation can easily surmount the tested repellents. Summarizing, both gels seem to have only an ineffective, non-permanent repellent effect. Nevertheless, only repellents reducing the number of birds using the treated structures by 100% are effective systems. Therefore the tested products are not recommendable for a successful feral pigeon management.

Systems based on exclusion and mechanical barriers still remain the most reliable repellents. However, the best way of efficiently coping with the pigeon problem in our cities seems to be the reduction of the pigeon population, and this can only be achieved by reducing the food supply of the birds [2].
